# Massive expansion of multiple clones in the mouse hematopoietic system long after whole-body X-irradiation

**DOI:** 10.1038/s41598-022-21621-6

**Published:** 2022-10-14

**Authors:** Kengo Yoshida, Yasunari Satoh, Arikuni Uchimura, Munechika Misumi, Seishi Kyoizumi, Masataka Taga, Yukiko Matsuda, Asao Noda, Yoichiro Kusunoki

**Affiliations:** 1 Department of Molecular Biosciences, Radiation Effects Research Foundation, 5-2 Hijiyama Park, Minami-Ku, Hiroshima, 732-0815 Japan; 2 Department of Statistics, Radiation Effects Research Foundation, Hiroshima, Japan

**Keywords:** Mutation, Haematopoiesis

## Abstract

Clonal hematopoiesis (CH) is prevalent in the elderly and associates with hematologic malignancy and cardiovascular disease. Although the risk of developing these diseases increases with radiation doses in atomic-bomb survivors, the causal relationship between radiation exposure and CH is unclear. This study investigated whether radiation exposure induces CH in mice 12–18 months after 3-Gy whole-body irradiation. We found radiation-associated increases in peripheral blood myeloid cells and red blood cell distribution width (RDW). Deep sequencing of bone marrow and non-hematopoietic tissue cells revealed recurrent somatic mutations specifically in the hematopoietic system in 11 of 12 irradiated mice but none in 6 non-irradiated mice. The irradiated mice possessed mutations with variant allele frequencies (VAFs) of > 0.02 on an average of 5.8 per mouse; mutations with VAFs of > 0.1 and/or deletion were prevalent. Examining hematopoietic stem/progenitor cells in two irradiated mice revealed several mutations co-existing in the same clones and multiple independent clones that deliver 60–80% of bone marrow nuclear cells. Our results indicate development of massive CH due to radiation exposure. Moreover, we have characterized mutations in radiation-induced CH.

## Introduction

As humans age, a limited number of hematopoietic stem/progenitor cells (HSPCs) in the bone marrow (BM) become prone to clonal expansion, resulting in the massive production of descendant blood cells into the periphery, a process known as clonal hematopoiesis (CH). CH occurs in at least 10–20% of people aged 70 years or older and links to increased risks of both hematologic malignancy and non-cancer diseases, including cardiovascular disease (CVD)^1,2^. The pathogenesis of CVD underlies the increased production of pro-inflammatory myeloid cells from clonally expanded HSPCs with mutations typically in *DNMT3A* and *TET2*^3–5^.

Theoretically, CH occurs ubiquitously in everyone by the age of 50^6^, but only a fraction of elderly individuals exhibit clonally expanded leukocyte populations at the widely accepted 0.02 variant allele frequency (VAF) threshold^1,4^. Thus, determinant factors that initiate or promote CH, particularly the cell-extrinsic factor of exposure to ionizing radiation at various doses, must also be assessed. In atomic-bomb survivors, clonal chromosomal aberrations were frequently detected in peripheral blood cells, suggesting that clonally expanded blood cells often appear following radiation exposure^7^. The number of peripheral blood monocytes, risk of CVD, and frequency of hematologic malignancy increased in proportion with the radiation dose to atomic-bomb survivors^8,9^. These findings suggest that radiation-related CH increases monocyte production and CVD risk. Studies of cancer radiotherapy demonstrated significant associations between radiation exposure and CH^10–12^, but the causal relationship and underlying mechanisms remain to be fully elucidated. Therefore, in this study, we evaluated the effects of radiation exposure on CH development and blood cell profiles in a mouse model. We characterized radiation-induced CH in mice, which may contribute to the understanding of the involvement of CH in the increased risk of hematologic malignancy and inflammatory non-cancer diseases among radiation-exposed individuals, long after radiation exposure.

## Methods

### Animals

We used (C57BL6 *HPRT*-dup-*GFP* × C3H/HeJ) F1 mice^13^, which are hybrids of female *HPRT*-dup-*GFP* and male C3H/HeJ (Japan SLC, Inc., Shizuoka, Japan) mice, to isolate in vivo* HPRT* mutant cells that could be detected by induction of GFP expression in a viable state. Forty-three mice (19 males and 24 females) were 3-Gy (Gy) whole-body irradiated at 6 weeks of age using an X-ray generator CP-160 (Faxitron Bioptics, Tucson, AZ, USA; 160 kV, 6.2 mA, 0.5 mm Al and 0.21 mm Cu filter; 56 keV actual energy) and an AE-1322 dosimeter (Ouyo Giken, Inc., Tokyo, Japan) at a dose rate of 0.8 Gy/min. Eleven mice (7 males and 4 females) were not irradiated and used as controls. The mice were housed in a specific pathogen-free facility at the Radiation Effects Research Foundation (RERF). We assumed that this relatively high dose (3 Gy) might efficiently induce somatic mutations and development of CH, considering a previous study^14^ wherein 2 Gy-irradiation induced chromosome aberrations in 10% of BM cells in (C57BL6 × C3H/HeJ) F1 mice; we expected a more efficient induction of mutation by irradiation with 3 Gy than that achieved with 2 Gy. Although these mice were used to characterize clonal cell populations with *HPRT* mutations, GFP expression levels did not significantly increase after irradiation (data not shown). Seven 3 Gy-irradiated mice and zero non-irradiated mice died (14.5 months, on an average, after irradiation) before the experimental analyses, leaving 36 irradiated and 11 non-irradiated mice for analysis. The BM cells were resuspended with fetal bovine serum (HyClone, Cytiva) containing 10% dimethyl sulfoxide in cryotubes and cryopreserved in liquid nitrogen. The cryopreserved cells were thawed quickly in a water bath at 37 °C, washed twice with cold RPMI1640 medium (Nacalai tesque, Inc., Kyoto, Japan) containing 10% fetal bovine serum, and used for DNA extraction or sorting experiments. The mouse experiments were approved by the Experimental Animal Care Committee at the RERF (No. 2020-01) and were performed in accordance with the 1996 National Institutes of Health guidelines and the RERF and ARRIVE guidelines for animal experiments.

### Blood cell profiling

Blood cell profiles were assessed using venous blood samples collected at 12–18 months post 0- or 3 Gy whole-body irradiation. The red blood cell distribution width (RDW) was measured using a hematological autoanalyzer XN-1000 (Sysmex, Kobe, Japan). We enumerated the lymphocytes (non-myeloid cells; defined as CD41^−^ CD11b^−^), granulocytes (CD41^−^ CD11b^+^ Ly6C^low^), and Ly6C-high monocytes (CD41^−^ CD11b^+^ Ly6C^high^) after excluding dead cells stained with 1 μg/mL DAPI (Invitrogen), using a MACSQuant X flow cytometer (Miltenyi Biotec, Bergisch Gladbach, Germany) and the FlowJo software (TreeStar, Ashland, OR, USA) as shown in Supplementary Fig. S1. Corresponding antibodies were purchased from BioLegend (San Diego, CA, USA; catalog numbers 101212 for APC CD11b, 133918 for PerCP-cyanine 5.5 CD41, and 128007 for PE Ly6C).

### Sequencing analysis

We performed whole-exome sequencing with deep coverage (500×) to identify CH with recurrent somatic mutations (VAF > 0.02) using BM cells from irradiated male mice (N = 12). Among these mice, eight and four mice exhibited higher (20.9–15.1) and lower (14.8–14.2) RDW values, respectively, and were selected for sequencing analysis to investigate whether CH develops in irradiated mice with higher RDWs. Control male mice (N = 7) were also subjected to sequencing analysis. DNA was extracted from BM cells from the femur using a QIAamp DNA Blood Mini Kit (Qiagen, Hilden, Germany) and sent to Macrogen, Inc. (Seoul, Korea) for library preparation using SureSelect XT Reagents (Agilent Technologies, Santa Clara, CA, USA) and sequencing on an Illumina NextSeq platform (San Diego, CA, USA). Sequence reads (fastq) were mapped using the Burrows–Wheeler alignment-maximal exact match algorithm to a mouse reference genome (mm10), after which the reads were sorted and indexed using SAMtools^15,16^. Duplicate reads of polymerase chain reaction amplicons were identified and removed using the MarkDuplicates (Picard) tool^17^. Single-nucleotide variants (SNVs) or small insertions or deletions were identified using MuTect2, a GATK4 package^17^, using a non-irradiated control mouse as a reference. To confirm the results, variant calling was performed using our previous results of whole-genome sequencing of young adult C57BL6/C3H F1 mice (Biosample IDs, SAMD00164355, SAMD00164356, SAMD00164357, SAMD00164358, SAMD00164359, and SAMD00164360, which were registered in the DDBJ Sequence Read Archive; https://www.ddbj.nig.ac.jp/dra/index-e.html) as another reference. Suspected germline variants were excluded based on a high VAF of > 0.35. That is, only variants with a VAF of 0.02–0.35 were further evaluated^1,4^. Variants supported by ≤ 5 reads or detected in most mice examined were filtered out to reduce artifacts. The effects of genetic variants were annotated and predicted using SnpEff^18^.

The frequencies of the detected somatic mutations were validated using the remaining DNA from BM cells by performing targeted amplicon sequencing (Illumina MiSeq, average depth of 30,000) with primers that amplified regions containing the mutated sequences. The Nextera XT Index Kit (Illumina) was used to attach dual indices and sequencing adaptors. The tissue specificities of the somatic mutations were also assessed in the BM, spleen, thymus, tail, kidneys, liver, brain, thyroid gland, and testes by performing targeted sequencing of DNA from these tissues.

### Cell sorting and HSPC colony formation

Using preserved BM cells isolated from two male mice, the mutation frequencies were determined in CD3^+^ T cells, CD19^+^ B cells, Gr-1^+^ granulocytes, and Ter119^+^ erythroid populations. The mutation frequencies were also determined in hematopoietic colonies derived from sorting single lineage^−^ Sca-1^+^ c-Kit^+^ CD34^−^ CD150^+^ CD48^−^ long-term hematopoietic stem cells (HSCs) and lineage^−^ Sca-1^+^ c-Kit^+^ CD34^+^ multipotent progenitors (MPPs) into 96-well plates (Supplementary Fig. S2)^19,20^. These cells were allowed to form colonies in MethoCult FG M3434 medium (STEMCELL Technologies, Vancouver, Canada) at 37 °C and 5% CO_2_ for 14 days. Appropriate antibodies were purchased from BioLegend (catalog numbers 100308 for PE CD3, 115519 for PE-cyanine7 CD19, 103432 for APC-cyanine7 CD48, 115922 for PerCP-cyanine5.5 CD150, 127623 for APC-cyanine7 Gr-1, 108108 for PE Sca-1, and 116222 for PE-cyanine7 Ter119) or Invitrogen (Carlsbad, CA, USA; 25-0031-82 for PE-cyanine7 CD3, 17-0193-80 for APC CD19, 11-0341-82 for FITC CD34, 17-1171-83 for APC c-Kit, and 25-5931-82 for PE-cyanine7 Gr-1), and the cells were sorted after excluding dead cells stained with DAPI, using a FACSAria II cell sorter (BD Biosciences, Franklin Lakes, NJ, USA). The frequencies of somatic mutations were examined by performing targeted amplicon sequencing.

### Statistical analysis

The primary outcome was the comparison of the prevalence of CH (specifically, the number of recurrent somatic mutations) between the two groups of irradiated and non-irradiated mice. The difference in the mutation prevalence between the two groups was tested for each non-CH mosaic, CH-associated mosaic, or CH-associated non-mosaic mutation (deletions, SNVs, and multisite mutations) based on an exact Poisson regression using SAS 9.4 (SAS Institute, Inc., Cary, NC, USA). In addition, multisite mutations were defined as clustered SNVs and insertions or deletions^21^. Other variables, including the lymphocyte and myeloid cell percentages, RDW, and their associations with irradiation were analyzed using linear regression with adjustment for the age (months old at measurement) and sex of the mice. Regression analyses were conducted using R version 4.1.2^22^, by graphically checking the heteroskedasticity in each response variable with the residuals. The Breusch–Pagan test was performed for each regression analysis. *P*-values were determined based on multiple linear regression, with a weighted least squares method employed by defining the weights such that the observations with lower variance were given more weight.

## Results

### Blood cell profiles

**Figure 1:**
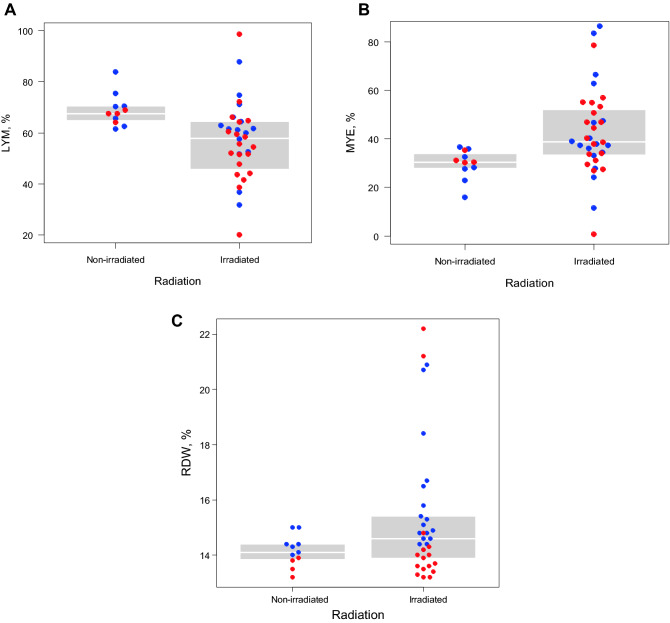
Blood cell profiles in mice 12–18 months post-irradiation. (**A**) Lymphocytes (LYM) were enumerated using flow cytometry as CD41^−^ and CD11b^−^ cells in the peripheral blood leukocytes and compared between radiation-exposed (N = 36) and non-exposed (N = 11) mice. The LYM percentages were significantly different (*P* < 0.01) based on linear-regression analysis after adjusting for age and sex. The blue and red dots represent male and female animals, respectively. Inter-quartile ranges are shown with gray-shaded areas. (**B**) Myeloid cell percentages (MYE, %) in the peripheral blood leukocytes were evaluated with CD41^−^ and CD11b^+^ populations, including Ly6C-low granulocytes and Ly6C-high monocytes. The same statistical analysis indicated a significant difference between radiation-exposed (N = 36) and non-exposed (N = 11) mice (*P* < 0.01). (**C**) RDW values of peripheral blood erythrocytes were measured using a hematological autoanalyzer in mice after irradiation. Three data were missing for irradiated mice because of sampling errors. The RDW values significantly differed between radiation-exposed (N = 33) and non-exposed (N = 11) mice (*P* < 0.01). RDW, red blood cell distribution width.

We evaluated irradiation-associated changes in blood cells in mice whole-body irradiated with 3 Gy of X-rays at 6 weeks of age. Representative results of blood cell profiling via flow cytometry are shown in Supplementary Fig. S1. The percentages of CD41^−^ CD11b^−^ lymphocytes (non-myeloid cells) declined, whereas those of myeloid cells (granulocytes plus monocytes) increased in irradiated mice at approximately 16 months after 3 Gy irradiation (18 months of age) compared with those in non-irradiated mice of the same age (Fig. 1A,B; *P* < 0.01 for both). The RDW values of peripheral erythrocytes, which are known to be relevant for the risks of hematologic and cardiovascular diseases and cancer^23^ as well as for CH prevalence^1^, also increased in mice at 16 months after 3 Gy irradiation (Fig. 1C; *P* < 0.01).

### Frequencies of recurrent somatic mutations in the BM

**Figure 2:**
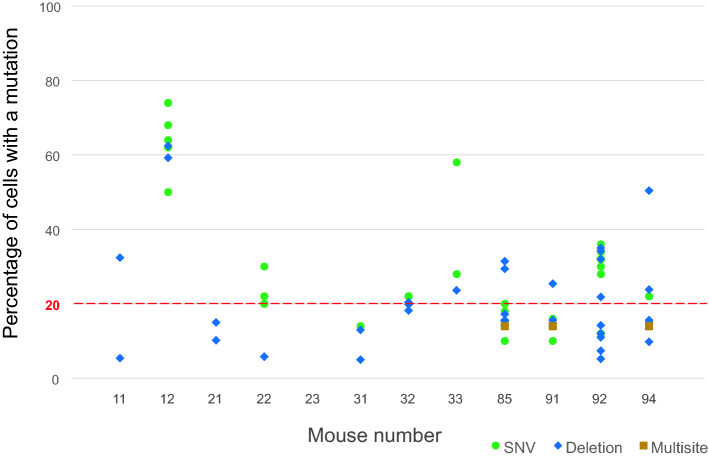
Clonal cell percentages in BM cells of irradiated mice. Cell percentages were estimated by doubling the VAFs of CH-associated mutations (N = 70, values are shown in Supplementary Table S1) in the BM because a somatic mutation normally occurs in a chromosome of a diploid cell. A VAF of 0.1 is equivalent to 20% of the cells. Multisite mutations are clustered SNVs and insertions/deletions. BM, bone marrow; CH, clonal hematopoiesis; SNV, single-nucleotide variant; VAF, variant allele frequency.

We examined recurrent somatic mutations, particularly those with a VAF of 0.02–0.35, in 12 irradiated mice and 6 non-irradiated control mice. Recurrent mutations were detected using whole-exome sequencing of BM cells at a high coverage depth (500×). Candidate somatic mutations were validated through targeted amplicon sequencing of DNA from the BM, spleen, tail, brain, and testes. 18 mice possessed 79 somatic mutations (Table 1). The genes harboring these mutations are shown in Supplementary Table S1. We classified the 79 somatic mutations into the following three groups (Table 1): (1) Nine mutations were detected in both hematopoietic and non-hematopoietic tissues with similar VAFs (suggesting mosaic mutations originated during the early stages of embryogenesis but not through massive expansion in the hematopoietic system); (2) Five mosaic mutations were present with low VAFs in non-hematopoietic tissues, such as the brain and testes, but were substantially expanded in the hematopoietic system (BM and spleen); and (3) The remaining 65 mutations were found only in the hematopoietic system. Mutations in the first group did not differ in number between the irradiated and control mice (*P* = 0.2), whereas the latter two groups considered as CH-associated mutations were observed exclusively in irradiated mice and the differences between irradiated and control mice were statistically suggestive (*P* = 0.1) and significant (*P* < 0.01) for the second and third groups of mutations, respectively. 11 of 12 irradiated mice possessed CH-associated mutations, and nine had mutations with VAF > 0.1, that is, clonal cell proportions of > 20% in the BM (Fig. 2), indicating 3 Gy radiation strongly enhanced CH development. However, the number or VAFs (either on average or at a maximum in each irradiated mouse) of CH-associated mutations was not significantly correlated with hematologic indicators such as the lymphocyte or myeloid cell percentages and RDW.

**Table 1:** Number of recurrent somatic mutations detected in 3 Gy-irradiated and non-irradiated mice.

Mouse	Age (months)	Number of recurrent mutations with VAF > 0.02 (with VAF > 0.1)			Tumors found at autopsy
		Mosaic^a^ not CH	CH-associated		
			Mosaic^a^	Non-mosaic	
**Irradiated**					
11	19.5	1 (1)	0	2 (1)	Liver, stomach
12	19.5	0	2 (2)	5 (5)	Liver
21	19.1	1 (0)	0	2 (0)	Liver
22	19.1	0	0	5 (4)	
23	19.1	1 (1)	0	0	Liver
31	19.1	0	0	3 (0)	Liver
32	19.1	1 (1)	0	5 (4)	
33	19.1	0	1 (1)	2 (2)	
85	16	0	0	10 (3)	
91	14.7	0	0	8 (1)	
92	14.7	0	1 (1)	15 (9)	
94	14.7	0	1 (1)	8 (2)	
Mean	17.8	0.3	0.4	5.4	
**Non-irradiated**					
51	19.1	1 (1)	0	0	
52	19.1	0	0	0	
53	19.4	1 (1)	0	0	Liver
61	19.2	0	0	0	
71	17.2	3 (3)	0	0	
73	18.5	0	0	0	
Mean	18.8	0.8	0	0	
Total		9 (8)	5 (5)	65 (31)	

^a^Mosaic mutations present in non-hematopoietic tissues. Each mouse (numbers 23 and 94) had one mosaic deletion; all other mutations were single-nucleotide variations. VAF, variant allele frequency.

### Characteristics of recurrent somatic mutations

Whereas mutations that arose during early development and expanded to both hematopoietic and non-hematopoietic tissues were not ascribed to radiation exposure, those observed only in the hematopoietic system potentially included radiation-induced mutations. The 65 hematopoietic-specific mutations (shown in Table 1) included 27 base substitutions, 35 deletions, and 3 multisite mutations, with the former two increasing significantly in irradiated mice (*P* < 0.01 for both, Table 2). The distributions of different types of single-nucleotide substitutions were similar to a large human population^1^ and a mouse study^24^, with a cytosine-to-thymine (C→T) transition as the most common change (Supplementary Fig. S3). In contrast, previous human CH studies reported considerably fewer cases of deletions and multisite mutations^1,2^. Of the 35 deletions, 12 had 1–3 nucleotide deletions; the remaining 23 deletions had 5–31 nucleotide deletions and included 8 deletions showing microhomology (at least two nucleotides) between the deleted and flanking sequences (Supplementary Table S1). Such deletions and multisite mutations are consistent with those characteristically observed in radiation-exposed mice and humans^21,25–27^.

**Table 2:** Number of clonal hematopoiesis (CH)-associated non-mosaic mutations (VAF > 0.02) separated by mutation types.

Mouse	Mutation types		
	Deletion	SNV	Multisite^a^
**Irradiated**			
11	2	0	0
12	2	3	0
21	2	0	0
22	1	4	0
23	0	0	0
31	2	1	0
32	3	2	0
33	1	1	0
85	6	3	1
91	3	4	1
92	9	6	0
94	4	3	1
Total	35	27	3

^a^Multisite mutations are clustered SNVs and insertions/deletions. SNV, single-nucleotide variation; VAF, variant allele frequency.

### Recurrent somatic mutations in blood cell lineages and HSPC colonies

**Figure 3:**
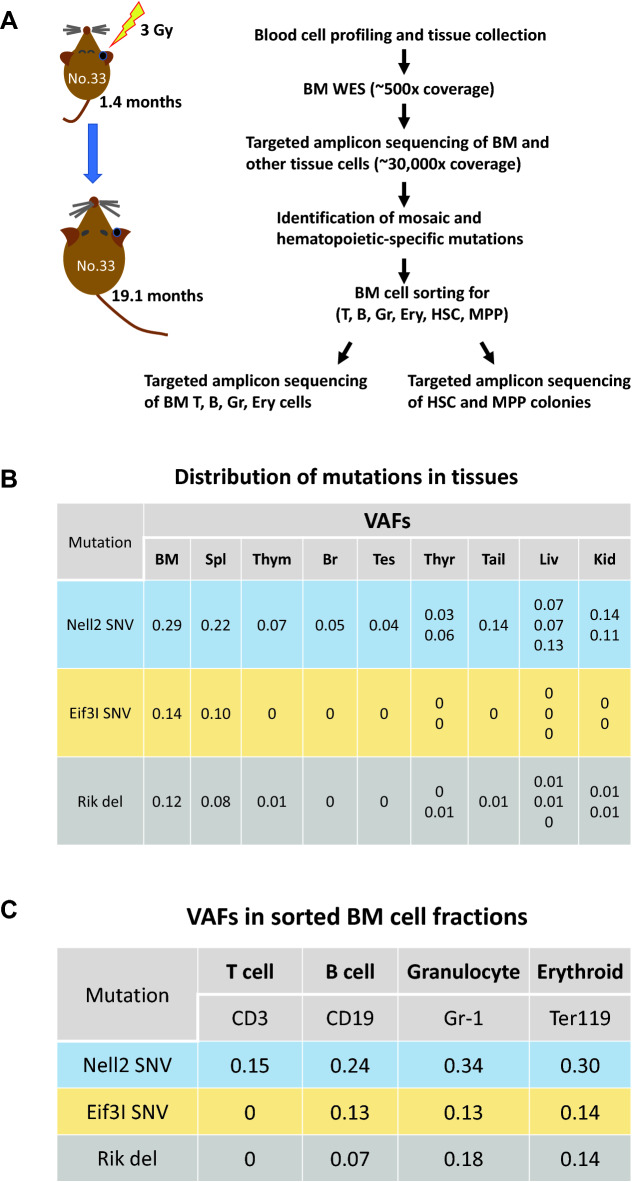

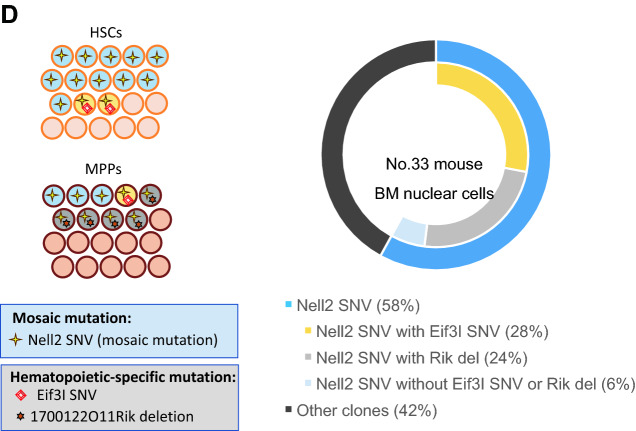
Distribution of CH-associated mutations in several tissues, BM blood cell types, and HSPC colonies in an irradiated mouse (mouse 33). (**A**) Schematic representation of experimental procedures for CH-associated mutations. (**B**) Nell2 SNV was considered to develop during early embryogenesis based on the VAFs of several non-hematopoietic organs, brain (Br), testes (Tes), and thyroid gland (Thyr). The Nell2 SNV was also prevalent in other non-hematopoietic blood-filtering tissues, tail, liver (Liv), and kidneys (Kid), BM, and lymphoid tissues, spleen (Spl) and thymus (Thym). Two separate sections for Thyr and Kid, three for Liv, and one for the other tissues were examined. Both the Eif3I SNV and 1700122O11Rik deletion (Rik del) showed limited distributions in these tissues (except for Spl). (**C**) In BM cells, the frequency of the Nell2 SNV was higher in B cells, granulocytes, and erythroid cells than in T cells. Both the Eif3I SNV and Rik del were frequent in BM cells, particularly in B cells, granulocytes, and erythroid cells. (**D**) Simplified illustration (left panel) shows coexistence of the Eif3I SNV and 1700122O11Rik del with the Nell2 SNV in hematopoietic colonies from HSCs and MPPs. Right panel depicts estimated proportions of clonal populations defined by recurrent mutations in BM nuclear cells of mouse 33, after irradiation. Clonal cell percentages were estimated by doubling the VAFs of the mutation in the BM, and their inclusion relationships were derived from the distribution of the three clonal mutations in HSPC colonies. BM, bone marrow; CH, clonal hematopoiesis; HSPC, hematopoietic stem/progenitor cell; SNV, single-nucleotide variant; VAF, variant allele frequency.

In irradiated mice, although the number of recurrent mutations per mouse varied widely from 2 to 16, some mutations found in the same mouse exhibited similar VAF values in each tissue examined (Supplementary Table S1), indicating that they appeared in the same cell population. To investigate the mutations’ origins and distribution in blood cell lineages, we selected two mice (numbers 33 and 85) without malignant or non-malignant symptoms and analyzed the mutation frequencies in T-cell, B-cell, granulocyte, and erythroid cell fractions that had been sorted from their BM, as well as in HSPC colonies obtained from single-cell sorted HSCs and MPPs of the same BM cells (Fig. 3A). In addition, overall, 68% of sorted HSC/MPPs produced colonies, and 40–52 colonies from each mouse (56% of the colonies) were sequenced.

To investigate how a mosaic mutation expanded between blood cell lineages as a result of CH development, we evaluated the VAFs in the T-cell, B-cell, granulocyte, or erythroid cell lineages and in 20 HSC or 20 MPP colonies in mouse 33 with three recurrent mutations, including one mosaic mutation (Supplementary Table S2). An SNV located at nucleotide position 95,432,868 of chromosome (chr) 15 (Nell2 gene) was considered to originate during early embryogenesis because it appeared in non-hematopoietic tissues, such as the brain, testes, and thyroid gland (Fig. 3B). Its VAF of 0.29 in the BM indicated that the embryonic mutant clone accounted for 58% of BM nuclear cells. Among the BM cell subsets of this mouse, the Nell2 SNV with VAF 0.15, 0.24, 0.34, or 0.30 appeared in T-cell, B-cell, granulocyte, or erythroid cell lineages, respectively (Fig. 3C); the VAF was approximately 0.5 in 13 of 20 HSC-derived clones and in 9 of 20 MPP-derived clones analyzed (Fig. 3D and Supplementary Table S2). Two other mutations—an SNV at position 79,076,783 of chr 15 (Eif3I gene) and deletion at position 48,036,757 of chr 17 (1700122O11Rik)—were specifically found in hematopoietic cells, with VAFs 0.14 and 0.12 in the BM, respectively, and VAFs close to 0.5 in mutually different cell clones from HSCs or MPPs (Fig. 3D, left panel). These findings indicate that cells carrying the Eif3I SNV and 1700122O11Rik deletion were two independent subpopulations within the cell population carrying the Nell2 SNV (Fig. 3D, right panel). In addition, the Nell2 SNV existed in T cells at the same level as in non-hematopoietic tissues and expanded in B and myeloid cells, whereas the other two mutations showed different distributions in B cells, granulocytes, and erythroid cells but were not detected in T cells or non-hematopoietic tissues (Fig. 3B,C).

**Figure 4:**
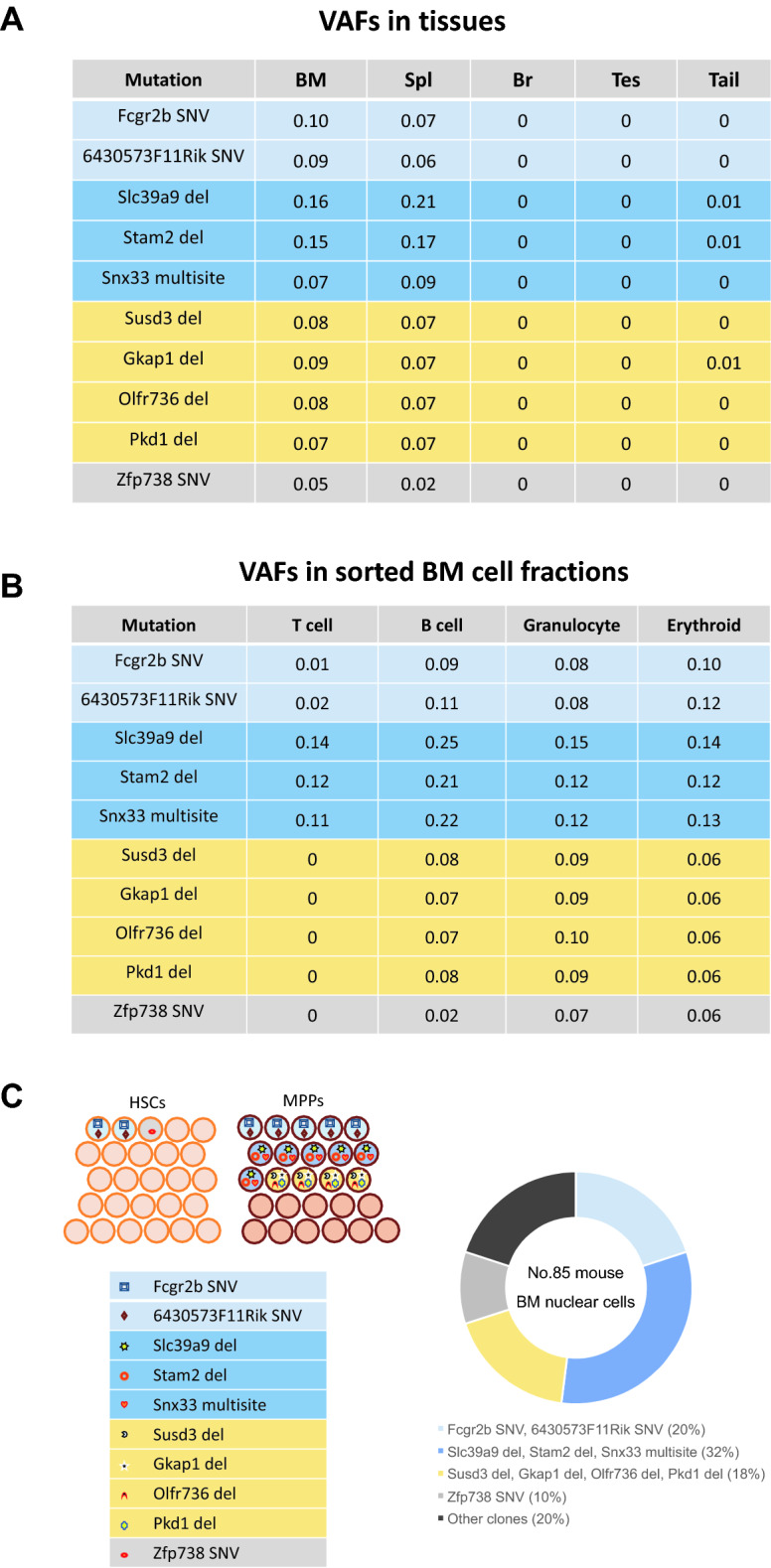
Distribution of CH-associated mutations in several tissues and BM blood cell types in a second irradiated mouse (mouse 85). This mouse was irradiated in a similar manner as was mouse 33 but was evaluated for CH prevalence at the age of 16 months. (**A**) All indicated mutations were undetectable in the brain and testes and rarely in the tail. In contrast, they were detected frequently in the BM and spleen, indicating hematopoietic specificity. (**B**) VAFs of these mutations were 0.02–0.25 in BM B cells, granulocytes, and erythroid cells. However, some were undetectable or scarce in BM T cells. (**C**) Several mutations coexisted in identical hematopoietic colonies from BM HSCs and MPPs, as indicated by the same colors (left panel); these coexisting mutations were distributed in different cell lineages with similar frequencies (also shown in **A** and **B**). Right panel depicts proportions of clonal populations estimated by doubling the VAFs of the mutation in the BM, and their exclusion relationships were derived from the distribution of the clones with different sets of mutations in HSPC colonies. BM, bone marrow; CH, clonal hematopoiesis; HSPC, hematopoietic stem/progenitor cell; MPP, multipotent progenitor; VAF, variant allele frequency.

In mouse 85 bearing 10 recurrent mutations, we assessed the VAFs of mutations in blood cell lineages and in 26 HSC or 26 MPP colonies to exemplify how several CH-associated mutations co-exist in the same clones (Supplementary Table S2). These mutations were all hematopoietic, with VAFs of 0.05–0.16 in whole BM nuclear cells (Fig. 4A), although five mutations were not detected in T cells (Fig. 4B). Evaluation of the VAFs in these HSC and MPP colonies indicated that at least four different HSPC populations independently expanded with two SNVs (located at chr 1, position 170,961,340 [Fcgr2b] and chr 8, position 36,512,373 [6430573F11Rik]), three mutations (chr 12, position 80,649,329 [Slc39a9]; chr 2, position 52,720,990 [Stam2]; chr 9, position 56,918,333 [Snx33]), four mutations (chr 13, position 49,239,072 [Susd3]; chr 13, position 58,259,599 [Gkap1]; chr 14, position 50,392,765 [Olfr736]; chr 17, position 24,594,785 [Pkd1]), or one mutation (chr 13, position 67,673,456 [Zfp738]) (Fig. 4C, left panel). The coexistence of the mutations in the same HSPC colonies likely reflects multiple independent HSPC clones that expanded following radiation injury, collectively comprising 80% of the whole BM nuclear cell population (Fig. 4C, right panel).

## Discussion

We detected recurrent somatic mutations (VAF > 0.02) almost exclusively in the hematopoietic tissues of irradiated mice at 16 months after 3 Gy irradiation but not in non-irradiated mice at the same age (Table 1). Most mutations exhibited characteristics of radiation-induced molecular changes, clearly demonstrating that radiation exposure induces the development of CH-associated mutations in mice; CH expansion is difficult to analyze in mice because of their short lifespan^24^. In addition, 3 Gy-irradiated mice exhibited radiation-associated increases in both their peripheral blood myeloid cell numbers and RDW values, which are often observed in survivors exposed to atomic-bomb radiation and in humans with CH^1,8,28^.

The CH observed in this study showed two notable characteristics. First, 3 Gy-irradiated mice had 5.8 mutations with a VAF of > 0.02 on average, approximately half of which showed a VAF of > 0.1, suggesting massive CH expansion. This is comparable to the VAFs of 0.12–0.14 in aging-associated CH in humans^1,2^ but is notably higher than the VAF of < 0.01 in non-irradiated mice^24^.

Second, HSC and MPP colony analyses in two irradiated mice (numbers 33 and 85) showed multiple mutations coexisting in the same colonies from HSPCs in different differentiation stages (Figs. 3D and 4C and Supplementary Table S2). Mouse 33 had two independent clones, one in both HSC and MPP clones and the other in MPP but not in HSC clones, which collectively accounted for 50% of its BM nuclear cells. Mouse 85 showed four independent clones, one in both HSC and MPP clones, another in one HSC clone, and the other two in MPP clones but not in HSC clones or T cells, which accounted for 80% of its BM nuclear cells. Such a vast expansion of multiple clones with independent mutations has been very recently observed in a few elderly people^29^. In addition, possible radiation-induced mutations (deletions and multisite mutations) were prevalent in MPP but not in HSC clones (Figs. 3D and 4C), and most of these mutations expanded in blood cell populations other than T cells (Figs. 3C and 4B). Although the number of hematopoietic clones analyzed was limited, it can be hypothesized that MPPs mutated by radiation predominantly expand to reconstitute radiation-injured hematopoiesis. This hypothesis may be, to some extent, supported by a previous study in which the output of HSCs to MPPs was enhanced following 5-fluoruracil-induced leukopenia^30^.

The CH-associated mutations identified included no advantageous mutations reported in aging-associated human CH^1,2,31^, such as *DNMT3A*, *TET2*, *ASXL1*, *TP53*, *JAK2*, and *PPM1D*, nor mutations described in CH cases treated with cancer radiotherapy^12^; therefore, the mutations identified in this study may not directly contribute to promoting CH. However, CH without known driver mutations is prevalent and such mutant hematopoietic clones grow at rates similar to those of clones with CH driver mutations^32^. Thus, the 22 CH-associated mutations identified in this study (Supplementary Table S1, mutations with predicted high or moderate impact) may functionally impact and potentially confer a selective advantage during stress and long-term hematopoiesis after radiation exposure. Another plausible mechanism underlying CH promotion due to radiation exposure is that cells with less deleterious or neutral mutations may have been selected for long-term hematopoiesis. Alternatively, neutral drift in CH development may play a key role in hematopoietic recovery from radiation injury. In neutral drift, some cells without a proliferative advantage can expand as a random event and such a stochastic effect becomes stronger as the size of the cell pool reduces^2^. A previous study revealed that 3 Gy radiation exposure reduced the HSPC pool to approximately 30%^33^, suggesting subsequent HSPC proliferation through neutral drift toward CH development^2^. Examining the longitudinal trajectory of a clonal mutation from its emergence to expansion or extinction would reveal how radiation-induced mutations contribute to hematopoiesis after radiation exposure.

CH-associated gene mutations are involved in the pathogenesis of CVD^34^, and higher VAFs, typically > 0.1, in CH cases are closely associated with increased morbidity and mortality^35^. Increases in blood myeloid cell numbers and RDW are also strong predictors of prognosis and mortality due to various diseases, including cancer and CVD^23^, and have been observed in atomic-bomb survivors^8,28^. However, there was no significant association between VAFs of CH-associated mutations and these blood markers in the irradiated mice examined in this study. This is likely because the number of mice examined was too small to fully evaluate the effects of radiation-induced CH and to exclude or adjust effects of other pathophysiological processes, such as tissue-specific inflammations and aging, on these markers. Another limitation of this study is that the functions of clonally expanded cell populations were not assessed, which can be evaluated by combining mutation and transcriptome analyses of mutated HSPCs at the single-cell level. Through precise assessments of mutations and their functions using an increased number of samples, future studies on massive CH in irradiated mice can determine the molecular mechanisms linking radiation exposure, CH, and enhanced inflammation during the development of cancer and non-cancer diseases.

The Nell2 SNV, an early embryogenic SNV mutation identified in this study, was expanded massively in hematopoietic cells; however, when this expansion occurred remains unknown. We recently developed a method for reconstructing early embryonic cell lineages using spontaneous de novo mutations that are detected directly using deep-coverage whole-genome sequencing of adult tissues to reconstruct the lineages based on the relationship of VAFs between mosaic mutations^36^. Using this method, we revealed that one spontaneous mutation occurs per cell division in early mouse embryos (before gastrulation) and that its distribution varies between embryonic tissues after gastrulation. The Nell2 SNV expansion in HSPCs may have occurred before irradiation. However, the VAF of this SNV in the thymus was as small as in non-hematopoietic organs (Fig. 3B), and that in T cells was lower than in the other blood cells examined (Fig. 3C), suggesting that expanded HSPCs did not greatly contribute to T-cell reconstitution after radiation injury. Therefore, this SNV likely expanded in HSPCs after irradiation and distributed largely to B and myeloid cells, although studies on the VAF before and after irradiation are needed to confirm this hypothesis. Notably, cell lineage tracking at single-cell-division resolution is useful for detecting endogenous genomic markers to evaluate clonal cell expansion in various tissues other than the hematopoietic system, which would reveal detailed information on radiation-induced clonal cell expansion and subsequent cancer and non-cancer diseases.

## Supplementary Information

Supplementary Figures.

Supplementary Tables.

## Data Availability

Data supporting the conclusions are available in the manuscript and its supplementary information. The raw sequencing data were registered at the DDBJ Sequencing Read Archive under accession number DRA013660. Source data are also provided with this paper.
