# Supplementary material for: Massive expansion of multiple clones in the mouse hematopoietic system long after whole-body X-irradiation
Source: Sci Rep. 2022 Oct 14;12:17276. doi: 10.1038/s41598-022-21621-6 (PMC9568546; doi:10.1038/s41598-022-21621-6)
Supplement: Supplementary file 1 — Supplementary Figures. [file 41598_2022_21621_MOESM1_ESM.docx]

**Massive expansion of multiple clones in the mouse hematopoietic system long after whole-body X-irradiation**

Kengo Yoshida, Yasunari Satoh, Arikuni Uchimura, Munechika Misumi, Seishi Kyoizumi, Masataka Taga, Yukiko Matsuda, Asao Noda, and Yoichiro Kusunoki

Figure S1

**A gating strategy for evaluating myeloid, lymphoid, and platelet (CD41+) populations in the peripheral blood.** A typical flow cytometry-based histogram (obtained after excluding doublets) is shown.

Figure S2

**Gating of HSC and MPP fractions for single-cell sorting after excluding dead cells and doublets.**

Figure S3

**The distributions of different types of single-nucleotide base-pair changes in CH-associated non-mosaic mutations (N = 27).**
